# Levels of active tyrosine kinase receptor determine the tumor response to Zalypsis

**DOI:** 10.1186/1471-2407-14-281

**Published:** 2014-04-23

**Authors:** Victoria Moneo, Beatriz G Serelde, Carmen Blanco-Aparicio, Ramon Diaz-Uriarte, Pablo Avilés, Gemma Santamaría, Juan C Tercero, Carmen Cuevas, Amancio Carnero

**Affiliations:** 1 PharmaMar R & D, Madrid, Spain; 2 Experimental Therapeutics Programme, Spanish National Cancer Center (CNIO), Madrid, Spain; 3 Structural Biology and Biocomputing Programme, Spanish National Cancer Center (CNIO), Madrid, Spain; 4 Instituto de Biomedicina de Sevilla, IBIS/Hospital Universitario Virgen del Rocio/CSIC/Universidad de Sevilla, Sevilla, Spain; 5 Consejo Superior de Investigaciones Cientificas, Sevilla, Spain; 6 Instituto de Biomedicina de Sevilla, Hospital Universitario Virgen del Rocio, Edificio IBIS, Consejo Superior de Investigaciones Cientificas, Avda. Manuel Siurot s/n., Sevilla 41013, Spain

**Keywords:** Zalypsis, Marine compound, PDGFR, Tyrosine kinase receptors, Antitumor compound

## Abstract

**Background:**

Zalypsis® is a marine compound in phase II clinical trials for multiple myeloma, cervical and endometrial cancer, and Ewing’s sarcoma. However, the determinants of the response to Zalypsis are not well known. The identification of biomarkers for Zalypsis activity would also contribute to broaden the spectrum of tumors by selecting those patients more likely to respond to this therapy.

**Methods:**

Using *in vitro* drug sensitivity data coupled with a set of molecular data from a panel of sarcoma cell lines, we developed molecular signatures that predict sensitivity to Zalypsis*.* We verified these results in culture and *in vivo* xenograft studies.

**Results:**

Zalypsis resistance was dependent on the expression levels of PDGFRα or constitutive phosphorylation of c-Kit, indicating that the activation of tyrosine kinase receptors (TKRs) may determine resistance to Zalypsis. To validate our observation, we measured the levels of total and active (phosphorylated) forms of the RTKs PDGFRα/β, c-Kit, and EGFR in a new panel of diverse solid tumor cell lines and found that the IC50 to the drug correlated with RTK activation in this new panel. We further tested our predictions about Zalypsis determinants for response *in vivo* in xenograft models. All cells lines expressing low levels of RTK signaling were sensitive to Zalypsis *in vivo*, whereas all cell lines except two with high levels of RTK signaling were resistant to the drug.

**Conclusions:**

RTK activation might provide important signals to overcome the cytotoxicity of Zalypsis and should be taken into consideration in current and future clinical trials.

## Background

During the past 30 years, medical oncologists have focused on optimizing the outcome of cancer patients by developing new antitumoral agents and defining new prognostic factors as well as integrating more effective supportive care measures. However, clinical anticancer strategies indicate that conceptually active therapies benefit only a small proportion of patients; thus, a large cohort of patients must be exposed to these antitumoral treatments to obtain a benefit in only a fraction of them.

Pharmacogenomic studies are aimed at identifying predictive biomarkers that can help to define subpopulations of patients who will, or will not, benefit from a particular therapy. These molecular markers of a response to a specific drug are not exclusive to the so-called “Targeted Therapies” but also have been identified for widely used cytotoxic agents. Representative examples include the relationship between mRNA expression and response and survival using antifolates [1], beta tubulin III mRNA levels and response to tubulin-interacting agents [2], PTEN methylation and resistance to CPT-11 [3], and Ras oncogenic activation and resistance to EGFR-interacting agents [4].

Zalypsis® (PM00104) is a marine-derived compound that has shown cytotoxic activity against various human tumor cell lines both *in vitro* and *in vivo*, including cell lines resistant to other chemotherapeutic agents [5,6]. Zalypsis is a novel antineoplastic agent currently in phase II clinical development in endometrial and cervical cancer, multiple myeloma, and Ewing’s sarcoma (http://clinicaltrials.gov/ct2/show/NCT01222767).

Structurally, Zalypsis contains a similar chemical scaffold to trabectedin, differing in an additional appended ring [7]. It has been shown that the trabectedin chemical scaffold forms a covalent bond with DNA [8], and the appended ring has been proposed to directly interact with the nucleotide excision repair (NER) endonuclease XPG [9,10]. Trabectedin and Zalypsis exhibit overall similarities and sequence-specific differences in their DNA footprint properties [7]. *In vitro* data suggests that Zalypsis has DNA-binding properties, induces cell cycle arrest, and inhibits transcription, eventually leading to apoptosis [5,11]. Although the precise mechanism of action of this agent remains mostly unknown, there is increasing experimental data describing Zalypsis’ antitumoral activity [12,13]. The binding to the minor groove of DNA is the main event in the antitumoral activity of Zalypsis and results in stabilization of the DNA duplex [11], mimicking a inter-strand crosslink. Treatment of cells lines with Zalypsis leads to cell cycle delay in S phase, activation of the DNA damage checkpoint, and cell death. Additionally, *Schizosaccharomyces pombe* cells containing a RAD51 mutation were found to be extremely sensitive to Zalypsis, suggesting that the compound induces double-strand breaks (DSBs) [5]. Experiments in isogenic cell lines have indicated that the cytotoxic effect of this compound is independent of functional nucleotide excision repair system properties [7]. However, the DNA damage repair machinery is essential to overcoming Zalypsis-induced DNA damage, suggesting that this damage is mainly due to DSBs [5].

The aim of this study was to identify biomarkers defining the molecular basis of sensitivity/resistance to Zalypsis to assist in its clinical development. To this end, we used a panel of solid tumors, including low-passage cell lines from untreated sarcoma tumor samples [14]. Using this panel of low-passage tumor cell lines, we assessed sensitivity to Zalypsis and other drugs currently used in sarcoma treatment [15] and found well-defined differences in sensitivity to the drugs tested. We analyzed the relationship between the IC50 to Zalypsis in the panel of tumor cell lines and the expression of a large panel of molecular markers, observing significant relationships between the direct alterations of the markers and specific compounds. The most relevant finding was that the increased signaling from RTKs determines Zalypsis resistance *in vitro* and in xenograft models *in vivo*.

## Methods

### Cell lines and culture conditions

The panel was composed of commercial and in-house-generated cell lines from patients of soft tissue sarcomas. For the generation of new cell lines, sterile fragments from resected tumors were minced in culture medium and then disaggregated by 1–2 h incubation in collagenase (100 U/ml) at 37°C. After 24 h, the medium was changed to F-10 Ham (Gibco) supplemented with 1% Ultroser G (Biosepra). The cell lines generated were cultured in F-10 Ham supplemented with 1% Ultroser G. A673 cells were cultured in RPMI (Sigma) and SW872 in Leibovitz L-15 (Sigma). All media were supplemented with 10% FBS, fungizone, and penicillin/streptomycin. Once the cells became confluent, adherent cells were removed by trypsin treatment and seeded at 1/2 or 1/3 ratio with medium. Throughout the establishment of these cell lines, their phenotypic features were followed. Additionally, the cell lines were routinely checked for mycoplasma contamination (INVIVOGEN). All cell lines used were established immortal tumor cell lines.

For the newly created human cell lines from resected tumor tissue, approval from local ethics committee at Hospital Universitario Virgen del Rocio (Comite etico de investigacion Hospital Universitario Virgen Del Rocío) was obtained (PI2012-0085) and informed consent was obtained from patients.

### Cytotoxicity assessment

The compounds were tested using 96-well trays. Cells growing in a flask were harvested just before becoming confluent, counted using a hemocytometer, and diluted with media by adjusting the concentration to the required number of cells per 0.2 ml (volume for each well). The cells were then seeded in 96-well trays at a density between 1,000 and 4,000 cells/well, depending on the cell size. The cells were allowed to settle down and grow for 24 hours before adding the drugs, which were weighed and diluted with DMSO to a concentration of 10 mM. A “mother plate” with serial dilutions was prepared at 200X the final concentration in the culture; 11 different concentrations were tested at 1/3 dilution in a range from 10 μM to 0.1 nM. When necessary, for highly sensitive or highly resistant cell lines, a new mother plate was generated by decreasing or increasing two more concentrations, as required. The final concentration of DMSO in the tissue culture media did not exceed 0.5%. The appropriate volume of the compound solution (usually 2 μl) was added automatically (Beckman FX 96 tip) to the media to reach the final concentration for each drug. The medium was removed from the cells and replaced with 0.2 ml of medium dosed with drug. Each concentration was assayed in triplicate. Two sets of control wells were included in each plate, containing either medium without drug or medium with the same concentration of DMSO. A third control set was obtained with the untreated cells just before the addition of the drugs (seeding control, number of cells starting the culture). The cells were exposed to the drugs for 96 hours and then washed twice with phosphate-buffered saline before being fixed with 10% glutaraldehyde. The cells were washed twice, fixed with crystal violet 0.5% for 30 minutes, washed extensively, solubilized with 15% acetic acid, and absorbance measured at 595 nm. The value of cytotoxicity was given as an IC50 concentration, the concentration a particular drug needed to inhibit by 50% the proliferation of a cell line or kill 50% of a cell population.

### RT-PCR

Total RNA was collected using the TRI-REAGENT (Molecular Research Center, Inc.). RT was performed (Promega) with 1 μg of RNA following the manufacturer’s protocol. cDNA (1 μg) was used for PCR, and the amplified products were analyzed by electrophoresis on a 1% agarose gel. The PCR primers used and the length of the amplified product are shown in Additional file 1: Table S1.

### Western blot analysis

Whole-cell extracts were prepared from cells and processed as previously reported [16]. Briefly, the harvested cells were washed once in cold phosphate-buffered saline (PBS) and suspended in 1 ml lysis-buffer (50 mM Tris–HCl pH 7.5, 1% NP-40, 10% glycerol, 150 mM NaCl, 2 mM, and Complete protease inhibitor cocktail -Roche-). The protein content of the lysates was determined by the modified method of Bradford. Proteins were separated on 7.5% SDS-PAGE gels, transferred onto Immobilon-P membranes (Millipore), immunostained, and visualized using the ECL detection system (Amersham). The expression of different proteins was determined using the antibodies described in Additional file 2: Table S2.

### Statistical analysis

Univariate Cox models were used to analyze the correlation between the IC50 for the drug and the expression of each biomarker in the cell line panel. For each drug, we therefore performed 15 Cox regression analyses, one for each biomarker. The p-values obtained in the Cox regression analysis were used to determine the relevance of the biomarker for predicting the sensitivity to the drug.

### *In vivo* xenograft response to Zalypsis

The experimental research on mice performed in this work complied with institutional, national, and international guidelines for the welfare of animals and was approved by the local ethics committee (Comité Ético de Experimentación Animal(CEEA)/CEI HU Virgen Del Rocío/IBIS).

Four to six week-old athymic nu/nu mice (Harlan Sprague Dawley) were s.c. xenografted into their right flank with approx. 0.5-1 × 10^7^ cells in 0.2 ml of a mixture (50:50; v:v) of Matrigel basement membrane matrix (Beckton Dickinson, Franklin Lakes, NJ, USA) and serum-free medium. When the tumors reached approx. 150 mm^3^, the mice were randomly assigned into treatment or control groups. Zalypsis® was intravenously administered either in 3 consecutive weekly doses (0.9 mg/kg/day) or in 2 cycles of 5 consecutive daily doses (0.3 mg/kg/day). Control animals received an equal volume of vehicle. Caliper measurements of the tumor diameters were performed twice weekly, and the tumor volumes were calculated according to the following formula: (a·b)2/2, where a and b were the longest and shortest diameters, respectively. The animals were humanely euthanized, according to Institutional Animal Care and Use Committee of PharmaMar, Inc. (Cambridge, MA, USA) guidelines, when their tumors reached 3000 mm^3^ or if significant toxicity (e.g., severe body weight reduction) was observed. Differences in tumor volumes between the treated and control groups were evaluated using the unpaired t-test. Statistical significance was defined as p < 0.05. The statistical analyses were performed by LabCat v8.0 SP1 (Innovative Programming Associates, Inc. NJ, USA).

## Results

### Analysis of expression of biomarkers in the cell line panel

The expression of protein and mRNA levels from different genes were analyzed under basal conditions in the absence of the drug and correlated with the *in vitro* sensitivity after exposure to Zalypsis, trabectedin, and doxorubicin. The panel included 22 genes involved in tumor progression, cell adhesion, cell cycle control, and cell signaling: Apaf-1, APC, cdk4, c-Kit, cyclin D1, E-cadherin, MDM2, MLH-1, MSH-2, p14^ARF^, p15^INK4b^, p16^INK4a^, p21^cip1^, p27^kip1^, p53, p73, p85, PDGFRα, p60^src^, PTEN, pAKT, and β-catenin. Fourteen genes were analyzed by the presence or absence of mRNA, and the remaining genes were evaluated by the protein level (see Additional file 1: Table S1, Additional file 2: Table S2 and Additional file 3: Table S3 for indication). For the statistical analysis, the presence or absence of RNA of each biomarker in each cell line was scored as 1 or 0, respectively (see Additional file 3: Table S3). For proteins, intermediate values were included when some biological relevance had been described for different levels. Only 15 biomarkers showed differences among the different cell lines and were used for the statistical analyses.

### Sensitivity to Zalypsis, trabectedin, and doxorubicin of low-passaged sarcoma cell lines

The panel of low-passage human sarcoma cell lines was treated with different concentrations of Zalypsis, trabectedin, and doxorubicin. The values of sensitivity (IC50) to trabectedin and doxorubicin were determined for comparison. The IC_50_ values were calculated as an average of three independent experiments performed in triplicate (Table 1). Response to each drug varied largely between the most sensitive and most resistant cell lines. In fact, the median IC50 for doxorubicin was 232 nM (range 14 to >300), indicating at least a 20-fold difference in sensitivity in the cell lines from the studied panel. The cell line panel was highly sensitive to trabectedin, with a median IC50 of 0.7 nM (range 0.1 to >100, 1000-fold difference). According to a previous report [14], the value of 1 nM IC50 is considered as the cut-off to separate cell lines sensitive or resistant to trabectedin (Table 1).

**Table 1:** IC50±SD values (nM) of a panel of low-passaged sarcoma cell lines in response to trabectedin, Zalypsis, and doxorubicin

**Cell line**	**Tumor origin**	**Doxo (nM)**	**Trabectedin (nM)**	**Zalypsis (nM)**
**CNIO AW**	LIPOSARCOMA	45	0.7 ± 0.1	0.16 ± 0.02
**CNIO AX**	LIPOSARCOMA	44	0.7 ± 0.3	0.32 ± 0.15
**SW872**	LIPOSARCOMA	>300	0.5 ± 0.1	0.87 ± 0.53
**1455**	LIPOSARCOMA	>300	0.1 ± 0.02	0.34 ± 0.32
**CNIO AA**	LEYOMYOSARCOMA	21.5	0.4 ± 0.1	0.5 ± 0.13
**CNIO AY**	LEYOMYOSARCOMA	44	9 ± 0.3	2580 ± 1785
**CNIO AZ**	FIBROUS TUMOR	14	5 ± 0.3	870 ± 184.5
**CNIO BC**	MPNST	>300	>100	664.75 ± 450.5
**CNIO BB**	MPNST	232	>100	1008.35 ± 353
**A673**	EWING SARCOMA	50	1 ± 0.07	0.42 ± 0.39
**CNIO BJ**	OSTEOSARCOMA	>300	2 ± 0.32	103.2 ± 4.38
**CNIO BF**	OSTEOSARCOMA	15	0.3 ± 0.04	501.95 ± 9.83
**CNIO BP**	OSTEOSARCOMA	>300	0.3 ± 0.05	0.29 ± 0.10
**SAOS-2**	OSTEOSARCOMA	>300	0.11 ± 0.02	0.94 ± 0.74
**CNIO BG**	MIXOID FIBROSARC	22	0.3 ± 0.02	1200 ± 565.69
**CNIO BM**	HIBERNOMA	>300	10 ± 0.9	3473 ± 169.71
**CNIO BN**	FIBROHISTIOCITOMA	>300	>100	4750 ± 70.71
**CNIO CE**	RABDOMIOSARC	>300	>100	2401 ± 637.81
**CNIO BI**	GIST	50	0.1 ± 0.02	4933 ± 644.88

The data represent the average IC50 ± SD of three experiments performed independently. In the case of doxorubicin, only the average IC50 is shown. MPNST: Malignant peripheral nerve sheath tumor. GIST: Gastrointestinal stromal tumor.

Similarly, the cell line panel showed a broad range of responses to Zalypsis, with a median IC50 of 502 nM (range 0.16 to 4933). It is evident that the panel cell lines can be divided into two groups according to sensitivity to Zalypsis, sensitive lines with an IC50 below 1 nM and resistant lines with an IC50 above 100 nM, establishing a more than 100-fold difference between the sensitive and resistant groups. Although liposarcoma-derived cell lines are highly sensitive to trabectedin and Zalypsis, there is no tissue pattern for the response, and the sensitivity to Zalypsis did not correlate with that for trabectedin (Table 1). Both trabectedin and Zalypsis showed a pattern of sensitivity that was different from doxorubicin, which suggests no cross-resistance.

**Figure 1:**
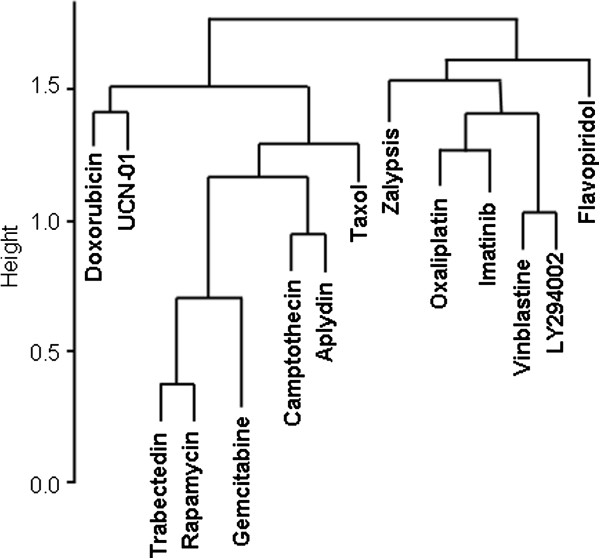
**Sensitivity homology tree.** We performed 15 Cox regression analyses, one for each marker and compared the relevance of each molecular marker to the responses to trabectedin, Zalypsis, and 12 other drugs. The multiple comparisons are expressed as a sensitivity homology tree view, which represent the similarity in the relevance of 15 different molecular markers with regard to a response to the drug.

To explore trabectedin and Zalypsis sensitivity further, we analyzed the relevance of each of the molecular markers for trabectedin and Zalypsis responses. To this end, we performed univariate Cox regression analyses to determine the relationship between sensitivity to Zalypsis (IC_50_) and the expression pattern of each marker across the cell line panel. Therefore, we performed 15 Cox regression analyses, one for each marker. We then compared the relevance of each molecular marker to the sensitivity to trabectedin or Zalypsis and compared with another 12 drugs previously analyzed [17]. The multiple comparisons were expressed as a sensitivity homology tree view (Figure 1), which represents the similarity in the relevance of 15 different molecular markers with regard to drug responses. According to the 15 markers analyzed, trabectedin and Zalypsis behave differently because they fall into different sensitivity homology tree branches. We can also observe that, although Zalypsis behaves similarly to oxaliplatin, trabectedin is closer to rapamycin or gemcitabine (Figure 1). Our sensitivity homology tree does not group the drugs by mechanism of action but by the relevance of certain markers to predict sensitivity or resistance, which clearly can be independent of the mechanism of action of each drug. Furthermore, Zalypsis and trabectedin also showed a different marker homology than doxorubicin, confirming the lack of cross-resistance *in vitro* and in patients previously described and indicating that they might be complementary to doxorubicin in treating solid tumors, especially sarcoma.

### Statistical analysis of biomarker expression correlation with sensitivity to Zalypsis

Next, we used the 15 independent univariate Cox regression analyses to determine the relationship between sensitivity to trabectedin or Zalypsis (IC_50_) and the expression pattern of each biomarker. As expected due to previous work [14], the p53 status was the main determinant of a trabectedin response in our cell line panel. However, we also found as secondary determinants PDGFRα and Cyclin D1, with statistically relevant predictive values.

**Figure 2:**
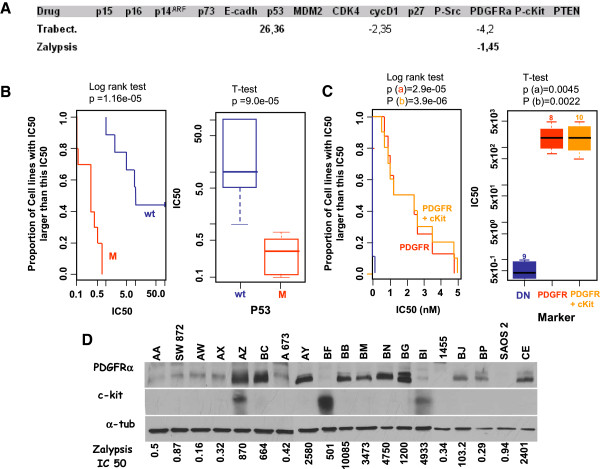
**Correlation of markers with trabectedin and Zalypsis cytotoxicity. A)** Statistically relevant (p < 0.005) correlations of molecular markers with trabectedin or Zalypsis treatment. The number indicates the correlation coefficient between the marker and the drug. **B and C)** Statistical graphs showing the correlation between individual markers and responses in the log rank test or Student t-test. The p value shows the statistical relevance of these correlations. **B)** Correlation of p53 mutations and the trabectedin IC50 (nM). M: mutant p53; WT: wild-type p53. **C)** Correlation of PDGFR/PDGFR + c-Kit and the Zalypsis IC50. **D)** Expression of PDGFRα and c-Kit proteins across the cell line panel and correlation with sensitivity (IC50 values) to Zalypsis.

We also found that the correlation of high PDGFRα expression and resistance to Zalypsis was the only relationship showing a statistically relevant value (p < 0.05) (Figure 2A and B). Cell lines with higher a IC50 for Zalypsis (AZ, BC, AY, BB, BM, BN, BG, and CE) showed higher levels of PDGFRα. Furthermore, BF (from osteosarcoma) and BI (from GIST), also resistant to Zalypsis, showed low PDGFRα levels but high levels of c-Kit (Figure 2C). A multivariate Cox regression analysis was also performed to explore the possibility of combining several markers to obtain a better predictive signature. In fact, the combined presence of high levels of c-Kit increased the statistical significance of the correlation (Figure 2B). None of the cells exhibited increased levels of EGFR (data not shown). We confirmed the cellular data for Zalypsis by comparing the cellular levels of PDGFRα and c-Kit with the IC50 for Zalypsis in each cell line (Figure 2C). These data suggest that the activation of either tyrosine kinase receptor could induce resistance to Zalypsis.

### Validation of the response to receptor tyrosine kinase activation

Our aim was to identify a predictive biomarker that allows the use of Zalypsis in a personalized manner and, if possible, to broaden the spectrum of tumors that can be treated with this drug. To validate the signature obtained in the sarcoma cell lines, we selected 9 new cell lines of other solid tumor origin, which, different from the low-passaged sarcoma cell lines, can also grow as xenografts and therefore can also be used for *in vivo* validation.

**Figure 3:**
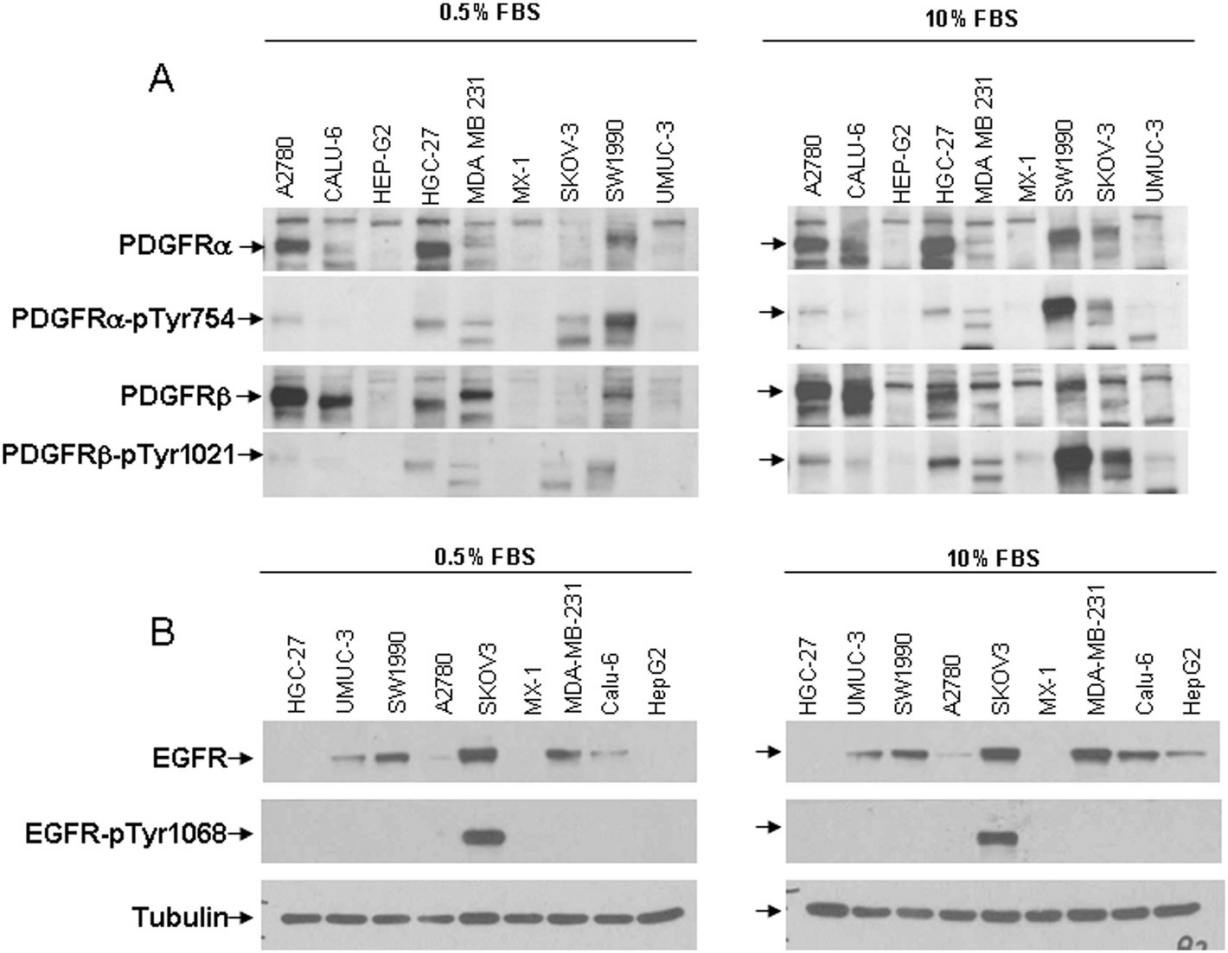
**Levels of total or phosphorylated PDGFRα/β receptors (A) or total or phosphorylated EGFR (B) in the nine solid tumor cell lines used for the *in vivo *experiments.** The data show the levels of proteins measured by western blotting.

**Figure 4:**
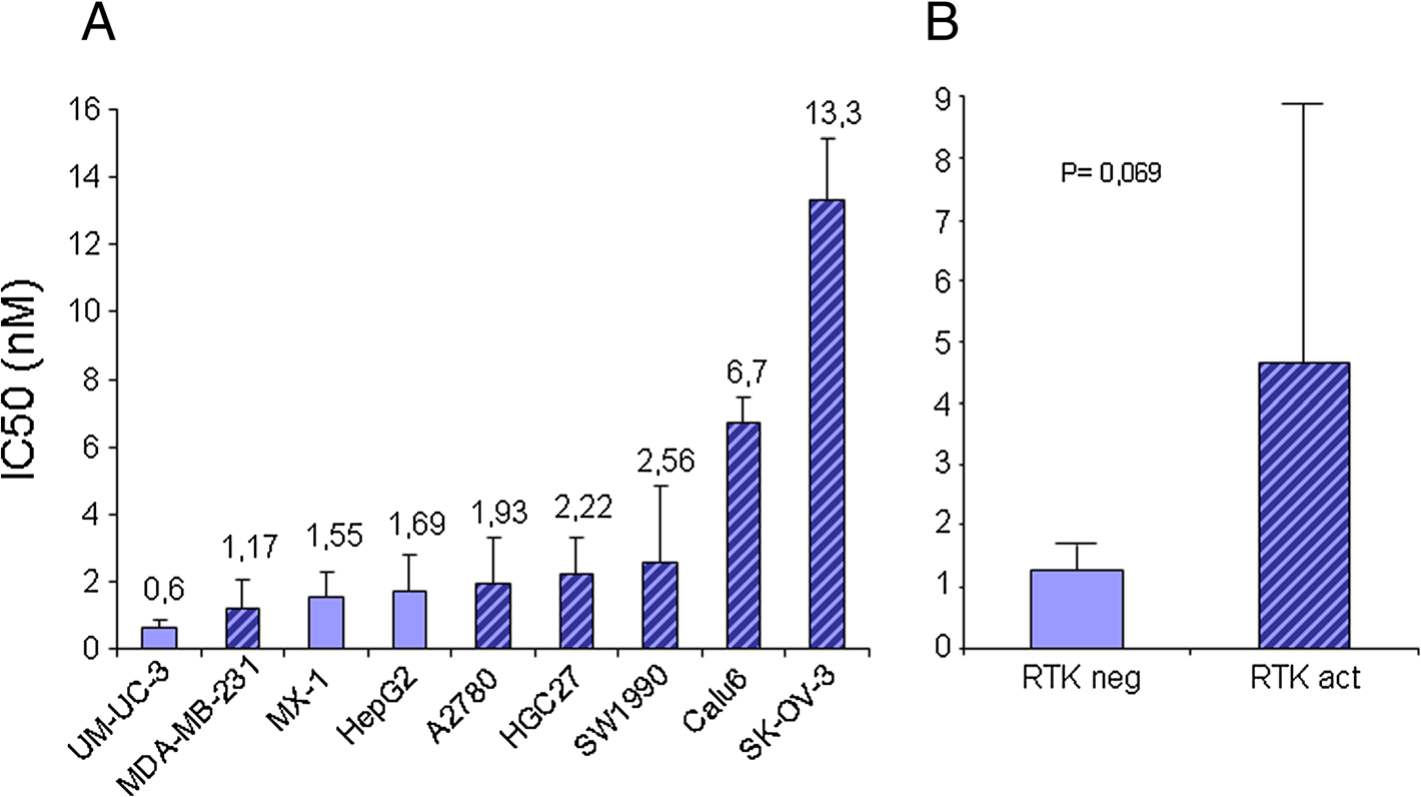
**Correlation of Zalipsis IC50 with RTK activation. A)** Graph showing the mean IC50 values for Zalypsis with respect to the average values for all IC50s. The bold bars indicate no constitutive activation of RTKs. The hatched bars indicate the activation of RTKs. The number above the bars shows the average IC50 values (nM) for the nine tumor cell lines. **B)** Graph comparing in groups the IC50 of cell lines with RTK activated or not activated. The Student T test was used to explore the statistical relevance.

The 9 cell lines were subjected to analysis to determine the levels of RTKs. To this end, we broadened the proteins analyzed and examined PDGFRα, PDGFRβ, EGFR, and c-Kit activation (measured as receptor phosphorylation) and the total levels at 10% or 0.5% serum (Figure 3). We found constitutive activation of the PDGFR signal, either α or β, in the A2780, Calu6, HGC27, and SW1990 cell lines and also in MDA-MB-231 cells. Furthermore, EGFR was constitutively activated in SKOV3 cells. None of the 9 cell lines showed c-Kit activation (data not shown). The IC50 for Zalypsis in the cell lines showed a rank from 13 nM to 0.6 nM, with SW1990, Calu6, and SKOV3 cells presenting an IC50 above the average. Figure 4A shows a comparison of the IC50 for Zalypsis with the constitutive activation of RTKs. Figure 4B shows a comparison of the IC50 of the cell lines with activated RTKs vs non-activated RTKs, with a borderline statistical significance (p = 0.069). As in the sarcoma cell line panel, the cell lines with constitutive RTK activation generally showed a higher IC50 for Zalypsis *in vitro* (Figure 4). However, the correlation did not occur in all cell lines because MDA-MB-231 cells had a low IC50, indicating that other factors may contribute to Zalypsis sensitivity in this cell line.

### *In vivo* response to Zalypsis in xenograft models

*In vitro* studies indicated that high levels of RTK phosphorylation and activation determined resistance to Zalypsis treatment. With the aim of validating this marker *in vivo*, we generated xenografts from the 9 cell lines that can be xenografted in mice. We used these cells because the low-passage sarcoma cells used in the predictive panel do not grow exponentially when xenografted in immunosuppressed mice. The xenografts of the different cell lines were treated with Zalypsis either at 0.3 mg/kg/day, qdx5x2. or 0.9 mg/kg/day, qdx7x3. A positive response was considered a reduction higher than 60% of the tumor burden with respect to the control, untreated tumor.

**Figure 5:**
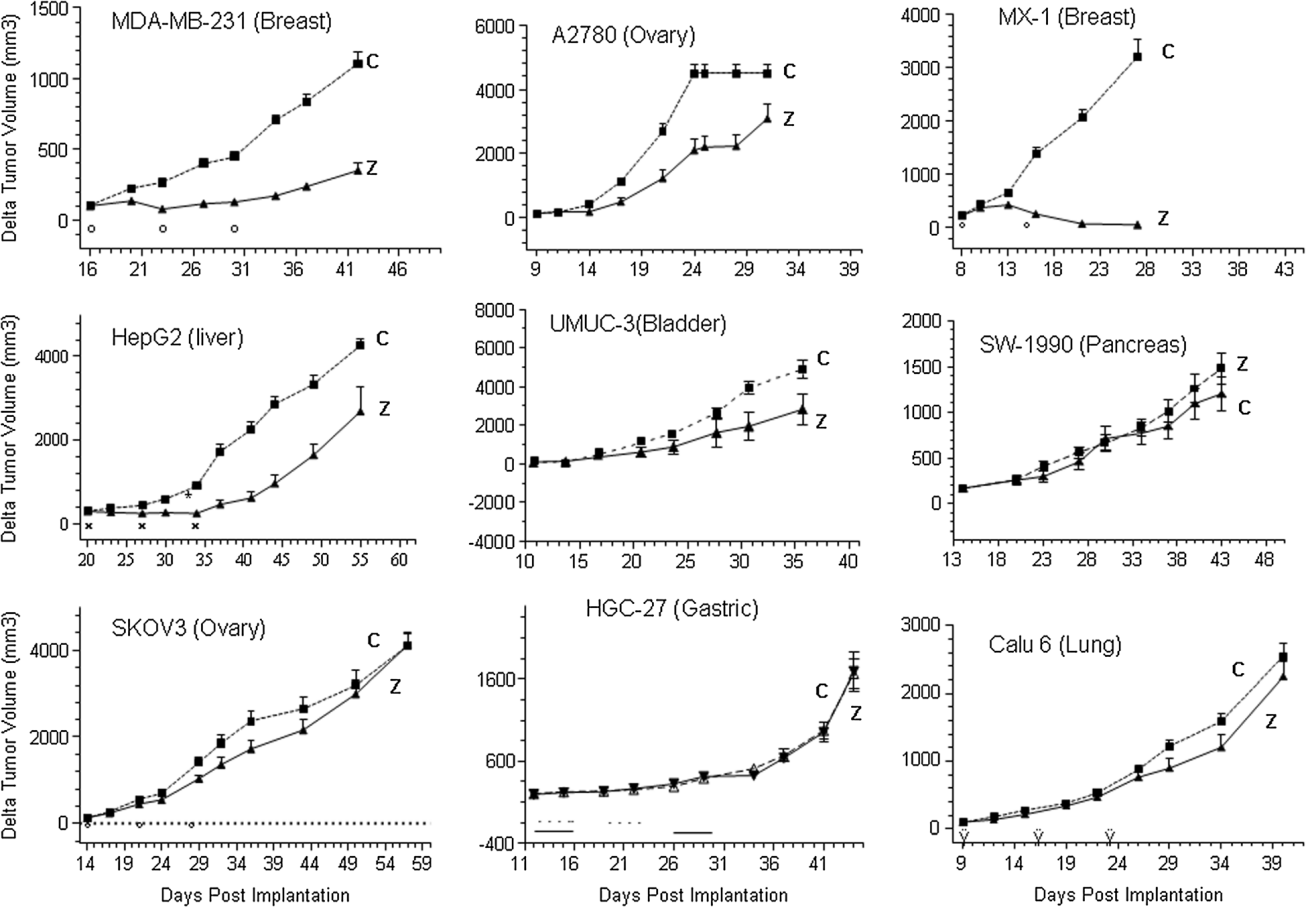
***In vivo *response of 9 carcinoma cell lines to Zalypsis treatment.** A total of 1 million cells were xenografted in nude mice. Once the tumors reached 100 mm^3^, Zalypsis treatment was initiated, as indicated. The tumors were measured weekly. (■, C: solvent-only treated mice) (▲, Z: Zalypsis-treated mice). Quintuplicate samples were used in each experiment.

Of the 9 cell line xenografts tested, 5 responded to treatment with Zalypsis, and 4 did not respond. The non-responding xenografts comprised CALU6, HGC27, SW1990, and SKOV-3 cells (Figure 5), all of which, except SKOV-3, showed high levels of phosphorylated PDGFRα/β (Figure 3A) under high (10%) or low (0.5%) serum conditions, indicating the constitutive expression of the receptor. Furthermore, SKOV-3 was the only cell line expressing high levels phosphorylated-EGFR, indicating a constitutively active EGF receptor (Figure 3B). A2780 and MDA-MB-231 cells, with increased levels of PDGFRα/β, still responded to Zalypsis treatment. The reason for this specific behavior for these cell lines is presently unclear and deserves further attention.

## Discussion

Compelling evidence from a number of laboratories has demonstrated the value of using biomarkers to select individual patients for targeted and non-targeted therapies [18,19]. The goal is to predict a response to chemotherapy to use agents in those patients more likely to respond, avoiding unnecessary toxicity. However, in most cases, the predictors are based on molecular signatures with low functional value *per se*. We developed a molecular signature based on the selection of molecular markers with functional relevance. The signatures obtained not only allow for a prediction of a response but also suggest possible mechanisms to overcoming resistance.

Zalypsis showed a different sensitivity profile from trabectedin in the cell line panel studied, indicating that alternative activities in cellular pathways or specific trends of intracellular metabolism might determine different activities. However, both marine compounds show effective results in liposarcoma- and fibrohistocytoma-derived cell lines, two types of cells very resistant to treatment including doxorubicin. In the remaining cases, the activity appears to be more dependent on the cell line than the tissue type. It is possible that one of the activities of these compounds is dependent on specific factors present in some sarcoma types. These specific factors may be the specific translocations that define certain sarcoma types; alternatively, the cell lineage-dependent genetic content may establish the sensitivity or resistance to a specific drug activity.

Furthermore, we also found that combinations of PDGFRα/β with other membrane receptors, such as EGFR or c-Kit, increase the predictability of the response to Zalypsis both *in vivo* and *in vitro*. RTK constitutive signaling might trigger the constitutive activation of the survival pathway through MEK or AKT activation, therefore accounting for the combined effect observed in our cell line panel both *in vivo* and *in vitro*. Active RTKs activate PI3K, leading to PDK1 and AKT activation [20]. Activated AKT can phosphorylate the pro-apoptotic Bcl-2 family member Bax at S184, inhibiting its conformational change and its subsequent translocation to mitochondria, thus preventing Bif-1 binding to Bax and alterations in mitochondrial membrane potential, cytochrome c release, caspase activation, and apoptosis [21-24]. Furthermore, Bcl-XL levels can be regulated by the PI3K pathway; upregulation of this protein implies survival, whereas downregulation leads to apoptosis. AKT also phosphorylates Foxo3a, inducing its mislocalization out of the nucleus and therefore inhibiting its proapoptotic activity [25]. Similarly, the phosphorylation of MDM2 by AKT induces its binding to p53 and the initiation of degradation, also acting on cellular survival [26]. However, RTK also activates the Ras pathway, leading to MEK and ERK activation, which also phosphorylates Bax to trigger a similar antiapoptotic response [27]. Additionally, as in the PI3K/AKT pathway, the Ras pathway can regulate the apoptotic response through IKK phosphorylation and the regulation of NFKB signaling [28].

RTK activation has been commonly linked to the resistance to anticancer therapies, either cytotoxic or targeted. Clearly, the upregulation of another receptor or its ligand, such as MET or HGF in lung cancer resistant to EGFR inhibitors, is a matter of concern in acquired resistance to RTK-targeted therapies [29-31]. Furthermore, TKR activation also has an important role in overcoming cytotoxicity to chemotherapy in different tumor types. For example, insulin-like growth factor-I receptor activation blocks doxorubicin cytotoxicity in sarcoma cells [32], and the EGFR inhibitor gefitinib sensitizes colon cancer cells to irinotecan [33]. Cisplatin-resistant neuroblastoma cells express enhanced levels of epidermal growth factor receptor (EGFR) and are sensitive to treatment with EGFR-specific inhibitors [34]. Other receptors such as TrkB protect neuroblastoma cells from chemotherapy-induced apoptosis via the phosphatidylinositol 3′-kinase pathway [35]. RTK inhibition is also effective in chemosensitizing human ovarian, nasopharyngeal, bladder, and neuroblastoma cancer cell lines, among others, when used in combination with cytotoxic agents [36-39]. Recently, we have also reported that SNPs in the PDGFRβ gene are related to increased levels of receptor and signaling, promoting chemotherapy resistance in colorectal cancer patients [40].

The logical conclusion is that the combination of Zalypsis with tyrosine kinase inhibitors would lead to a greater efficacy of treatment. Because the constitutive activation of c-Kit might also contribute to resistance, Zalypsis + imatinib may also be an interesting combination. There are several tyrosine kinase inhibitors approved with different specificities for different tyrosine kinases [41]. Imatinib and sunitinib also inhibit PDGFRα and c-Kit and other RTKs, such as VEGFR1 and FLT3, whereas sorafenib appears to be more specific for PDGFRβ but also inhibits c-Kit, FLT3, and VEGFR2. Nilotinib inhibits PDGFRα and -β and c-Kit. In contrast, gefitinib, erlotinib, and lapatinib are more specific for the EGFR family. We propose the use of a specific tyrosine kinase inhibitor according to the RTK active in the patient’s tumor, thus promoting personalized treatment.

However, further studies have to be conducted to fully validate this approach.

## Conclusions

We identified a molecular signature based on the combined activation of tyrosine kinase receptors that predicts resistance to Zalypsis in a broad panel of solid tumor cell lines. This signature was validated *in vivo* in xenograft assays. This signature deserves validation in tumor samples of patients treated with Zalypsis, with clinical trials currently ongoing, and also suggests a potential benefit for the combination of Zalypsis with tyrosine kinase inhibitors, which warrants further studies.

## Competing interests

VM, PA, GS, JCT, and CC are Pharmamar employees.

AC, BGS, CB-A, and RD-U declare no competing interests.

The sponsors had no role in the study design, in the collection, analysis, and interpretation of data, or in the writing of the manuscript and the decision to submit the manuscript for publication.

## Authors’ contributions

VM, PA, GS, BGS, and CB-A performed the experiments. RD-U performed the statistical analysis. CB-A, JCT, CC, and AC designed the experiments and analyzed the data. AC wrote the manuscript. CB-A, JCT, CC, and AC edited the manuscript. All authors read and approved the final manuscript.

## Pre-publication history

The pre-publication history for this paper can be accessed here:

http://www.biomedcentral.com/1471-2407/14/281/prepub

## Supplementary Material

Additional file 1: Table S1 Primers used to identify the mRNA expression of the different molecular markers used in this study. Click here for file

Additional file 2: Table S2 Antibodies used to identify the protein expression of the different molecular markers used in this study. Click here for file

Additional file 3: Table S3 Results of the presence (+) or absence (-) of different mRNAs in our cell line panel. The numbers identify the relative levels of the protein in each case. Click here for file
