# Supplementary material for: Levels of active tyrosine kinase receptor determine the tumor response to Zalypsis
Source: BMC Cancer. 2014 Apr 23;14:281. doi: 10.1186/1471-2407-14-281 (PMC4023704; doi:10.1186/1471-2407-14-281)
Supplement: Additional file 1: Table S1 — Primers used to identify the mRNA expression of the different molecular markers used in this study. [file 1471-2407-14-281-S1.doc]

**Additional file 1:** **Table S1:** Primers used to identify the mRNA expression of the different molecular markers used in this study.

| **Gene** | **Primers** | **Fragment size** |
| --- | --- | --- |
| **PTEN** | Oligo +: CAGAAAGACTTGAAGGCGTAT Tm 60  Oligo -: GTAACGGCTGAGGGAACTC Tm 60 | 620 bp |
| **P85** | Oligo +: AA GAA CAG TGC CAG ACC CAA Tm 60  Oligo -: ACT CGT TCA ACT TCT TTT GCC Tm 60 | 310 bp |
| **Apaf-1** | Oligo +: AGG ACA TCA AGA CAT CCT AC Tm 58  Oligo -: CAC ACA GGA CTG TCC TTA CA Tm 58 | 310 bp |
| **p16** | Oligo +: CAACGCACCGAATAGTTACG Tm 60  Oligo -: TACCGTGCGACATCGCGAT Tm 60 | 270 bp |
| **p15** | Oligo +: ATGCGCGAGGAGAACAAGGGC Tm 68  Oligo -: GGGCGGCTGGGGAACCTGG Tm 68 | 440 bp |
| **p21** | Oligo +: CATGTCAGAACCGGCTGGGGA Tm 68  Oligo -: CTTCCTCTGCTGTCCCCTGCA Tm 68 | 320 bp |
| **p27** | Oligo +: ATGTCAAACGTGCGAGTGTC Tm 60  Oligo -: TTACGTTTGACGTCTTCTGAG Tm 60 | 541 bp |
| **p14ARF** | Oligo +: GAGTGGCGCTGCTCACCTC Tm 60  Oligo -: TACCGTGCGACATCGCGAT Tm 60 | 510 bp |
| **E-cadherin** | Oligo +: TCCCATCAGCTGCCCAGAA Tm 60  Oligo -: ATGACTCCTGTGTTCCTGTTA Tm 60 | 500 bp |
| **p73** | Oligo +: TTTAACAGGATTGGGGTGTCC Tm62  Oligo -: CGTGAACTCCTCCTTGATGG Tm 62 | 410 bp |
| **APC** | Oligo +: GAATAGCCAGAATTCAGCAAATCG Tm 68  Oligo -: GAAAGTTTCATTAGAACACACACAG Tm 68 | 720 bp |
| **β-catenin** | Oligo +: AGCTGATTTGATGGAGTTGGA Tm60  Oligo -: GCTACTTGTTCTTGAGTGAAG Tm 60 | 240 bp |
| **MSH2** | Oligo +: AGAGATTGAATTTAGTGGAAGC TM 60  Oligo -: ACAATAGCTTATCAATATTACCTT TM 60 | 1777 bp |
| **MLH1** | Oligo +: GCATCTAGACGTTTCCTTGG TM 60  Oligo -: GAGGAATTGGAGCCCAGGA TM 60 | 367 bp |
| **β−actin** | Oligo +: AGGCCAACCGCGAGAAGATGAC Tm 70  Oligo -: GAAGTCCAGGGCGACGTAGCA Tm 68 | 330 bp |
