# Supplementary material for: Levels of active tyrosine kinase receptor determine the tumor response to Zalypsis
Source: BMC Cancer. 2014 Apr 23;14:281. doi: 10.1186/1471-2407-14-281 (PMC4023704; doi:10.1186/1471-2407-14-281)
Supplement: Additional file 2: Table S2 — Antibodies used to identify the protein expression of the different molecular markers used in this study. [file 1471-2407-14-281-S2.doc]

**Additional file 2: Table S2:** Antibodies used to identify the protein expression of the different molecular markers used in this study.

| **Primary Antibody** | **Supplier** | **Secondary Antibody** |
| --- | --- | --- |
| Anti-PDGFRα | Abcam (ab71009) | Anti-rabbit IgG (Calbiochem) |
| Anti-phospho-PDGFRα | Abcam (ab5460) | Anti-mouse IgG (Promega) |
| Anti-PDGFR**β** | Santa Cruz (sc-339) | Anti-rabbit IgG (Calbiochem) |
| Anti-phospho-PDGFR**β** | Santa Cruz (sc-12909-R) | Anti-mouse IgG (Promega) |
| Anti-EGFR | Abcam (ab2430) | Anti-rabbit IgG (Calbiochem) |
| Anti-phospho-EGFR | Abcam (ab5644) | Anti-mouse IgG (Promega) |
| Anti-c-Kit c-19 | Santa Cruz (sc-168) | Anti-rabbit IgG (Calbiochem) |
| Anti-phospho-c-Kit | Santa Cruz (sc-101659) | Anti-mouse IgG (Promega) |
| Anti-**β**-actin | Abcam (ab113279) | Anti-rabbit IgG (Calbiochem) |
| Anti-p53 FL393 | Santa Cruz (sc-6243) | Anti-rabbit IgG (Calbiochem) |
| Anti-MDM2 | Santa Cruz (sc-965) | Anti-mouse IgG (Promega) |
| Anti-CDK4 | Santa Cruz (sc-601) | Anti-rabbit IgG (Calbiochem) |
| Anti-cyclin D1 (clone EP12) | Dako (M3642) | Anti-mouse IgG (Promega) |
| Anti-p27 | Transduction laboratories (610241) | Anti-mouse IgG (Promega) |
| Anti-PTEN | Millipore (07-1372) | Anti-rabbit IgG (Calbiochem) |
| Anti-AKT | Cell Signaling (9272) | Anti-rabbit IgG (Calbiochem) |
| Anti-AKT-P Ser 473 | Cell Signaling (9271) | Anti-rabbit IgG (Calbiochem) |
