# Supplementary material for: Levels of active tyrosine kinase receptor determine the tumor response to Zalypsis
Source: BMC Cancer. 2014 Apr 23;14:281. doi: 10.1186/1471-2407-14-281 (PMC4023704; doi:10.1186/1471-2407-14-281)

**Additional file 3: Table S3**: Results of the presence (+) or absence (-) of different mRNAs in our cell line panel. The numbers identify the relative levels of the protein in each case.


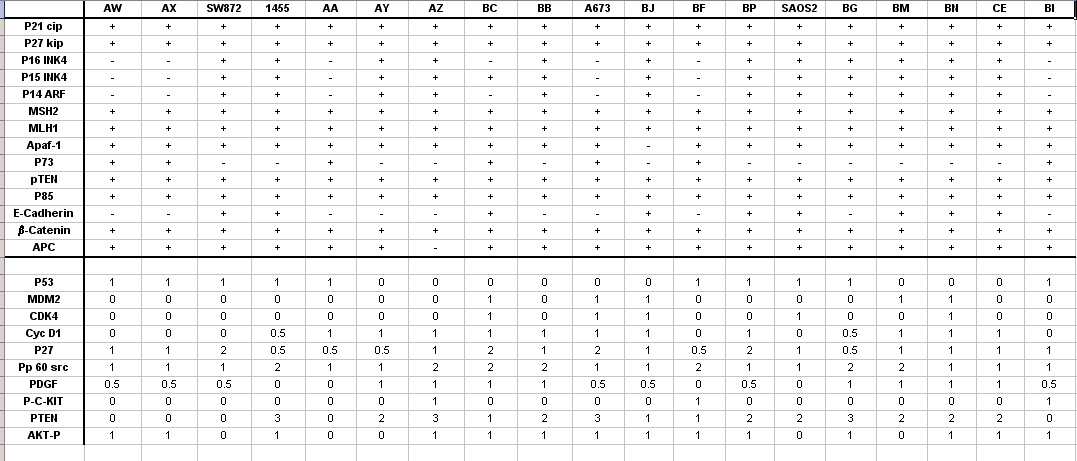

Supplement: Additional file 3: Table S3 — Results of the presence (+) or absence (-) of different mRNAs in our cell line panel. The numbers identify the relative levels of the protein in each case. [file 1471-2407-14-281-S3.doc]
